# Automatic Shuttlecock Fall Detection System in or out of a Court in Badminton Games—Challenges, Problems, and Solutions from a Practical Point of View

**DOI:** 10.3390/s22218098

**Published:** 2022-10-22

**Authors:** Michał Kopania, Jarosław Nowisz, Artur Przelaskowski

**Affiliations:** Faculty of Mathematics and Information Science, Warsaw University of Technology, Koszykowa 75, 00-662 Warszawa, Poland

**Keywords:** image processing, sports, motion capture, instant review system, challenge, badminton

## Abstract

We built an Instant Review System (IRS) for badminton, also named a Challenge System. It allows players to verify linesmen in/out decisions and makes the game fairer. Elements such as lighting, the influence of air-conditioning on the flight trajectory, or the moving mats can significantly impact the final in/out decision. Due to the construction of the shuttlecock, it behaves differently during the flight than, for example, a tennis ball. This publication discusses the problems we encountered during our work with the proposed solution. We present the evolution of the system’s architecture: the first version with the cameras mounted above the court and placed around the court close to the lines, tracking the shuttlecock in 3D; and the second, improved version with cameras placed only around the court, without 3D reconstruction. We used our system during the BWF World Senior Badminton Championships in Katowice. We present the system’s results from this tournament and compare them with linesmen’s decisions. We describe the system’s verification process by the Badminton World Federation and Polish Badminton Federation and discuss evaluation methods for such systems. Our solution is comparable to the commercial product used in the biggest badminton tournaments in regard to processing time and accuracy. Still, our architecture and algorithms make installing it much easier and faster, making the system more adaptive, reliable, flexible, and universal in relation to the practical requirements of sports halls.

## 1. Introduction

Badminton is the fastest sports game in the world. The shuttlecock can travel at a speed of over 400 km/h. The world record measured in a laboratory in 2013 is 493 km/h [1]. Because of such a high speed and human-eye perception, the line judges often cannot tell if the shuttlecock was in or out. Analyzing slow-motion video footage, a referee can judge if there was an out, but that process takes some time. The solution can be a computer vision system that automatically makes in/out decisions.

In this work, we present the results of theoretical, implementation, and experimental research on the location of the fall of a shuttlecock. The result of these studies is a working shuttlecock tracking system adapted to the conditions of the badminton game in a real-life scenario, in accordance with the applicable standards of the game and the time and quality requirements. We used our system at BWF World Senior Badminton Championships in Katowice, where over 1500 players competed on 12 courts. In this paper, we share our experience, the problems and challenges we encountered during building and operating our system, and how we solved them from a practical point of view.

Detection, segmentation, and tracking of objects are well-explored topics by researchers. Many algorithms have been developed, from simple shape and color segmentation methods to deep-learning algorithms. These algorithms are used in commercial systems such as Hawk-Eye [2], STATS SportVU [3], or ChyronHego TRACAB [4]. For example, TRACAB, which is mainly used during football games for tracking players, uses machine-learning algorithms and convolutional neural networks [5].

It should be noted that optical tracking data is prone to errors introduced by occlusions, which is why still, in existing systems, a human operator is required to correct these errors during measurement [6].

Still, offered systems are not 100% accurate—the Hawk-Eye’s claimed accuracy in tennis is 2.6 mm [7]. Despite the sometimes-erroneous decisions of the computer system, such systems are generally more accurate than humans [8]. Thus since 2001, computer vision applications have been used in many sports [9,10].

Just before Hawk-Eye’s system was adapted and used in badminton for the first time in 2014, there were theoretical proposals from researchers to build such a system specifically for badminton [11].

The main challenge for scientists and developers for systems supporting linesmen’s decisions is improving ball detection speed and accuracy. Compared to detecting larger objects, detecting fast-moving small objects, such as shuttlecocks, is still challenging. The performance is relatively low and requires many hardware resources [12].

Our first attempt to build an instant review system was similar to the Hawk-Eye system architecture in the measurement part (cameras placement). It used six cameras above the court mounted just below the ceiling of the hall and 14 cameras located around the court 1 m above the ground.

After tests, we removed the cameras above the court to simplify and shorten installation. We had to introduce a new algorithm to detect the moment of contact of the shuttle with the ground. The current version of our system does not calculate shuttlecock trajectories in 3D. Still, it can inform the umpire and the players if there was an out or not with comparable accuracy. It can be ready to operate within two or three hours.

## 2. Materials and Methods

### 2.1. Requirements, Problems, and Proposed Solution

The system works as follows. The number of cameras and their location is selected, so they see at least all the lines and areas around them and a flying shuttlecock approaching the ground. Video streams from cameras go to computers, where our software processes them and, by analyzing the shuttle trajectory, calculates where the shuttle hits the ground by comparing this place with the court model or previously detected court lines.

A player who disagrees with the linesman’s decision raises his hand and asks the umpire for a challenge. The umpire sends the challenge request to the system operator. He then starts the procedure and receives information from the system about whether the line judge has made a good decision or not. The operator or a designated judge may verify the system’s result and manually correct it. After verification, the system generates an animation and presents it to the players, judges, spectators, and TV broadcasters.

When designing the system, we adopted the following assumptions. The system should be as accurate as possible. The whole verification procedure should not take longer than 25 s. The installation time should be as short as possible, no longer than 4 h per court. The installation should not require more than two people. All equipment should take up as little space and weigh as little as possible.

Problems we faced during our research can be divided into two major groups: operational, meaning connected with external conditions; and algorithmic. Each mentioned problem will be described further with the proposed solution methods. Operational problems: lighting conditions, lack of space between courts for cameras, hardware, wiring, and power-related issues, securing the cameras, bent court lines, court mats deforming during the game, drift caused by air-conditioning, and equipment transportation. Algorithmic issues: camera calibration, court recognition, object detection and tracking, object segmentation, motion blur, finding shuttlecock ground hit position with respect to the court, occlusion of the tracked object, speed of algorithms, and flickering light.

### 2.2. Hardware Limitations, Wiring, and Data-Transfer-Related Issues

The system’s accuracy is highly dependent on the camera’s resolution and frame rate. We use cameras with a resolution of 800 × 600, with a maximum speed of 200 frames per second. When we started our project in 2017, it was the best that the market had to offer in accordance with our budget. Such a resolution proved to be sufficient to achieve satisfactory localization accuracy of the falling shuttlecock with moderate requirements for computational complexity.

The velocity of a shuttlecock approaching the ground depends on the initial speed, and distance traveled [13]. For smashes, it can be over 33 m/s. For high clear shots, it is just a terminal velocity of 6.7 m/s [14]. Using cameras operating with 100 fps gives us the distance a shuttlecock is moving between two consecutive frames, from about 70 to 330 mm (roughly ten times the diameter of the cork of the shuttlecock: 26–28 mm). Lighting conditions (described in the next section) and the lens and matrices available on the market limit possibilities to increase the shutter speed. We set up cameras in our system to 150 fps and use interpolation in our software to increase accuracy when the shuttle hits the ground between recorded frames. Alternatively, one can use more cameras mounted close to each other with shutters triggered at different timestamps and merge streams from them.

There are four primary interfaces (Table 1): GigE, CoaXPress, USB, and CameraLink to transfer data from cameras. Any WiFi or other radio-based communication with cameras is not feasible in crowded and prone-to-interference areas, such as sports halls during tournaments, so cables are the only option.

Designing hardware infrastructure is challenging. One can keep computers outside the playing zone and use longer GigE or CoaXPress cables or place the computer close to the cameras and use USB or CameraLink cables. It is also possible to use concentrators close to cameras and transfer data between concentrators and computers with optical fiber.

The market offer changes rapidly. Today’s cameras offer 2832 px × 2840 px resolution at 190 fps with CoaXPress 2.0 interface (2× CXP-12). When we started working on our system a few years ago, cameras with similar speeds had much smaller resolutions and offered a GigE interface.

We decided then to use GigE cables. They are relatively cheap, robust, and easy to cut and assemble. They can be used to transfer data and power the cameras by using Power over Ethernet technology (PoE). We keep our servers away from the courts and do not use any devices close to the cameras. This approach simplifies the installation of cameras between courts when the space is tight, but the cabling is sometimes tricky and time-consuming.

The amount of data transferred from cameras and the efficiency of algorithms detecting and tracking shuttlecock imply the necessary computing power and count of servers.

The essential factor is the expected response time of the system. We were surprised that the audience did not expect an immediate result. If the system responds within 10 s, the audience waits tensely for the visualization of where the shuttle has fallen, and this additional break is beneficial for the show. However, the system’s response cannot take longer than 25 s because the audience becomes bored after that. The times cited are our observations from over a dozen tournaments where we tested our system. Knowing those limitations, we can select the appropriate hardware for our algorithms. We use 8 servers and 15 cameras: 14 around the court and 1 with a broader view of the whole court. Each server supports two cameras. The operator uses the extra camera and server to monitor the system.

During the tournament, players and the TV crew walk around the court. They may step on the wire or even disconnect it from the camera. Connection-monitoring tools must be available for the operator, who should be warned when too many communication errors arise.

### 2.3. Lighting Conditions

Different sports venues have different lighting. In order to record video with a speed of over 150 fps, the lights must be strong enough. That problem does not exist in tennis (excluding evening matches), where we usually have daylight.

To deal with that issue, we tested different lenses and chose the one with the lowest F-number.

If the light is not bright enough, more extended exposition and higher camera-gain values are necessary. Extending exposition makes the motion blur effect more visible, and deblurring algorithms must be applied to reduce it. Increasing the gain causes higher image noise.

Since, as mentioned in the previous section, we use cameras with limited resolution, it is possible to upgrade them to newer models with much higher resolutions to increase the system’s accuracy if required. However, most of our algorithms and other hardware should also be corrected and adapted to the new quality of the signals. According to our rough estimates, the benefits are not obvious.

Our experiments measured how increasing gain affects PSNR and SNR values. We tested two sensors (Table 2) with the same lenses, acquired 20 images of size 4096 × 2160 pixels of the same static scene, and calculated the average PSNR and SNR values (Table 3).

Increasing the gain increases the noise, but increasing the gain up to +12 dB does not affect our object segmentation and tracking algorithms described in later sections.

Even in a hall with the poorest light conditions, we were able to acquire images with a speed of 150 frames per second, but only one place had strong enough lights installed to record a video with a speed of over 200 fps.

### 2.4. Securing the Cameras

It is infrequent in badminton for players to jump over advertising boards, so the cameras behind them are safe. Despite this, we plan to equip our cameras with safety cases. Such cases are used in volleyball, where it is common to knock over cameras with the ball or by the jumping player.

After positioning the cameras with respect to the court, they should not be moved. Still, the line judges or players may unintentionally move our cameras while entering the court.

Cameras cannot be permanently attached to the floor; it is better to destroy the camera than break the leg of a player who jumps on it.

Our software warns the operator when the camera is moved. The displacement is detected by comparing the reference image with the current image from the camera. Our software recalculates the camera’s position when the movement is slight, and the camera still sees the line. The operator must react and fix the camera’s position if the line is not visible.

### 2.5. Bent Court Lines

**Figure 1:**
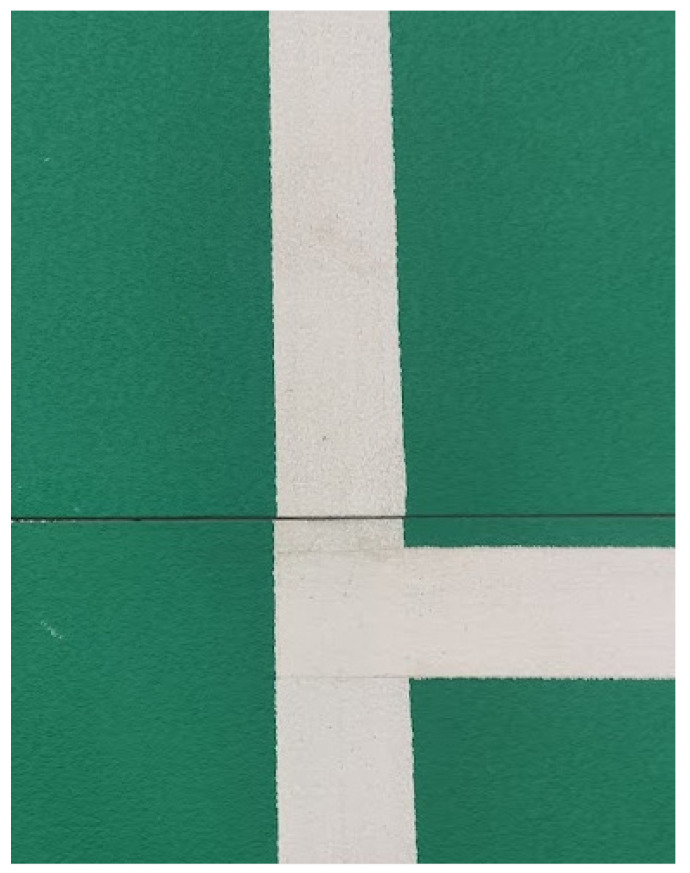
Imprecise mats connection, causing a 2 mm line shift.

Organizers of badminton tournaments place special rubber mats on each court. These mats consist of several pieces to facilitate their transport. The pieces are zipped together when put on the floor. Unfortunately, during installation, the parts of a mat may not be correctly assembled, and a slight shift may occur (Figure 1).

**Figure 2:**
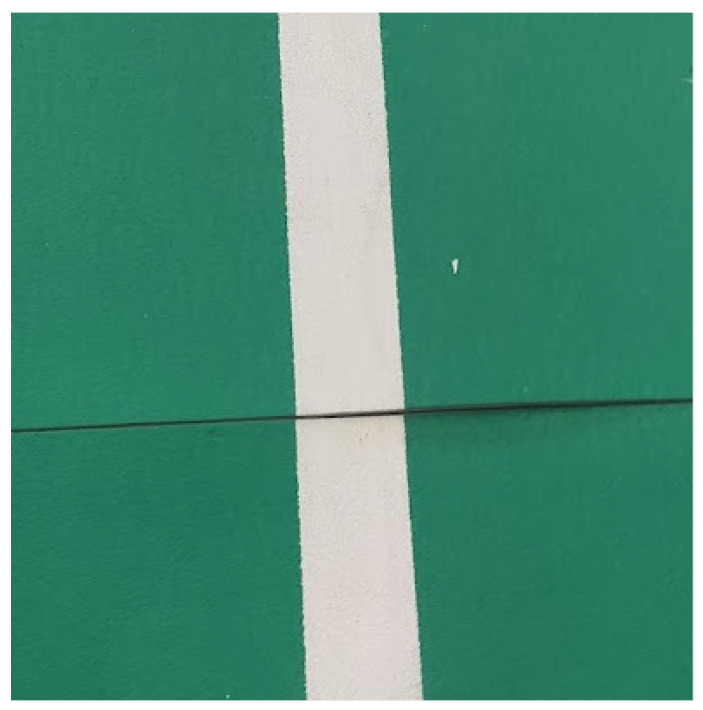
Deformation of a playing mat during the game.

Badminton is a high-speed game. Players rapidly change directions. Court mats are made from flex material to give additional amortization for the athletes. The drawback is that mats may deform during the game, and the court no longer fits the model (Figure 2). In fact, in a real-life situation, when the court deformation is significant enough to be spotted, the game is paused, and the court is fixed.

Our first version of the system used cameras above the court. The system had to recognize the court and transform it into a court model for further processing. We have learned from our experience that there are slight deviations in the actual court size from the dimensions prescribed by the regulations. It means that the court model defined with respect to the regulations cannot be used. Before each match, our system recognized the lines to create a precise model which matched the actual court.

The current version of our system uses only cameras located around the court, aimed at the court lines, and no court model is calculated. Instead, single lines are detected. They do not have to be perfectly straight for the system to work correctly.

### 2.6. Drift Caused by Air-Conditioning

A shuttlecock is a lightweight object (weighs 5 g), so it is easily affected by air drift. Equations that predict shuttlecock trajectory [15,16] do not consider that additional force. Moreover, the air currents’ temperature, direction, and strength change over time. For example, in the morning, when usually fewer spectators visit the venue, the air moves differently than when the hall is packed in the evening.

Instead of physical trajectory models, algorithms in the first system version have used adaptive methods such as the Kalman filter [17] as part of the tracking pipeline.

In the current system version, a camera only sees the shuttlecock in the last fragment of the trajectory when approaching the ground. We still use the Kalman filter for shuttlecock position interpolation.

### 2.7. Camera Calibration

The camera’s calibration is essential to achieve precise court model fitting when 3D tracking is involved.

We use zoom lenses to deal with different distances to the court lines. Zoom lenses have worse parameters than fixed-focal-length ones. Any change in lens settings requires camera recalibration. It is crucial that the calibration process is fast and easy to perform.

**Figure 3:**
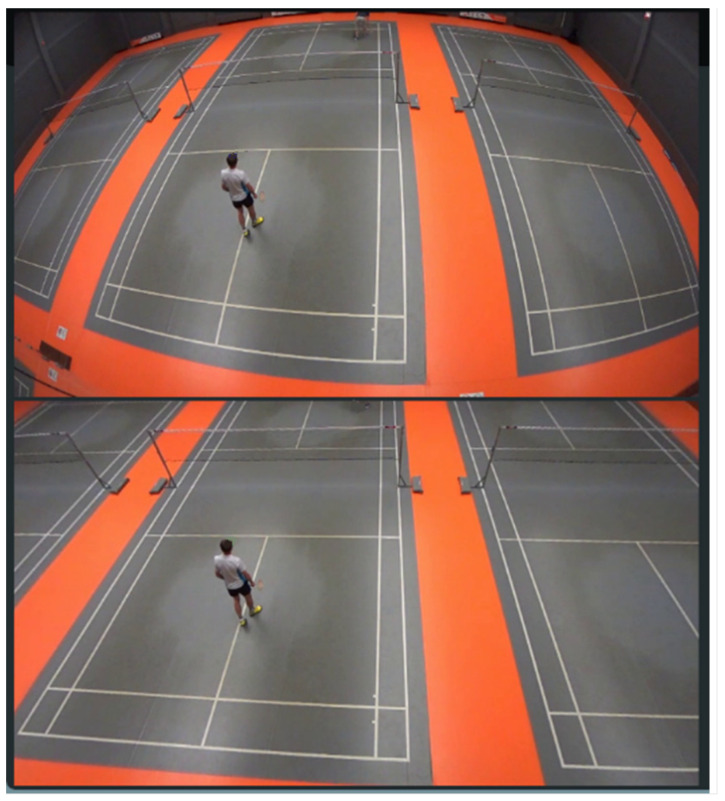
Original image (**upper**) and image after applying distortion coefficients calculated during the calibration process (**lower**).

Figure 3 shows an original image from the camera’s footage (upper) and the same image after applying distortion coefficients calculated during the calibration process.

Researchers widely investigated camera calibration and proposed many algorithms (see, for example, References [18,19]).

The calibration of cameras mounted high above the court is much more complex than calibration of cameras placed around the court, 1 m above the ground. Cameras from the first group are usually installed at least 15 m from the center of the court. Their depth of field starts from 5 m to infinity. A standard calibration board has dimensions of 1 m × 1 m or less, but for the best results, the markers should fill the whole image, which is impossible. Using more giant calibration boards is impractical because of transportation difficulties. Moreover, it is impossible to calibrate a camera that is already mounted because it is usually installed very high, and the light conditions are not good enough.

The cameras close to the court and the ground are much easier to calibrate. Their depth of field starts much closer to sensors. Thus, a standard 1 m × 1 m calibration board usually fills the whole image. The operator may calibrate cameras once installed in their final location. As mentioned earlier, for our algorithms working on streams from cameras close to the court, the accuracy of calibration is not essential. We can even use an automatic calibration process proposed by Daniel Santana-Cedrés et al. [20]. Our current system version uses only cameras close to the court, and we do not reconstruct 3D trajectories but instead detect the moment of and the place of the shuttlecock’s ground touch. For this algorithm, the court lines do not have to be ideally straight to produce correctly in/out decisions.

From our experiments, we learned that the following list of best practices could reduce reprojection error more than three times compared to the standard procedure [21]. We propose the following:

1. Adjust the shutter and gain to the lighting conditions.
2. Ensure that the lighting is even and that there are no overexposed or underexposed areas. Use a light diffuser if necessary.
3. Place the camera and the calibration board in the distance that fits into the depth of field.
4. Focus the camera. Use patches of regions around all detected markers. For each patch, calculate sharpness by using one of the methods proposed in Reference [22].
5. Choose the right size calibration target. It should cover as much as possible of the total area when seen parallel to the camera. Ideally, the markers should fill the whole image area.
6. Perform calibration at the working distance. The camera should be focused at this distance.
7. The calibration board should have a high feature count. A board with circles is better than a chessboard.
8. Collect images from 6 different areas and tilts: close parallel to the camera, far parallel to the camera, moved to the left and tilted 10–20 degrees, moved to the right and tilted 10–20 degrees, moved closer to the camera to the left and tilted 30–40 degrees, moved closer to the camera to the right, and tilted 30–40 degrees. The left and right positions must be symmetrical. Tilting more is not recommended as feature localization accuracy suffers.
9. Use a good calibration board. Boards printed by specialized companies are better than inkjet- or laser-printed ones. If one must use an inkjet-printed pattern, glue it to a flat and rigid surface; glass might be a good option (ensure to paint its back with black color to avoid lighting from the back through the glass and paper).
10. Mount the board vertically or flat on rigid support to minimize distortion and bow.
11. Consider moving the camera instead of the board. Use a quality tripod, and do not touch lenses.
12. Before starting the acquisition, make sure that the camera is warmed up.
13. Inspect reprojection errors, both per-view and per-feature. If any of these appear as outliers, acquire a new set of images and recalibrate.
14. Due to overfitting, obtaining a low reprojection error does not equal a good camera calibration but merely indicates that the provided data/evidence can be described with the used model. Parameter uncertainties are indications of how well the chosen camera model was constrained.
15. Analyze the individual reprojection errors. Their direction and magnitude should not correlate with position; that is, they should point chaotically in all directions.

### 2.8. Court Recognition

Our current system version detects only single lines, but the earlier version with cameras mounted above the court detects the whole court before matches. The court’s dimensions are standardized, so it is pretty easy to recognize the court and fit it into the model. Hough transform can be applied to the Sobel filter output to find court lines. Then the intersections of the lines can be detected by using the Bentley—Ottmann algorithm [23]. Another method is to use the Harris corner detector to detect court corners [24]. The implementation of a court recognition method proposed by Dirk Farin [25] can be found on GitHub [26].

We used a modified version [27] of the Mask R-CNN [28] to detect and segment the court from the image. The masks generated by the neural network are dilated before applying to the image to make sure that all court lines are visible. Then the Hough transform and pattern matching are applied to match the detected court with the model. We then analyzed the lines and corners fitting and adjust the model in case of inconsistencies caused by rubber court mat deformations described earlier in this paper.

**Figure 4:**
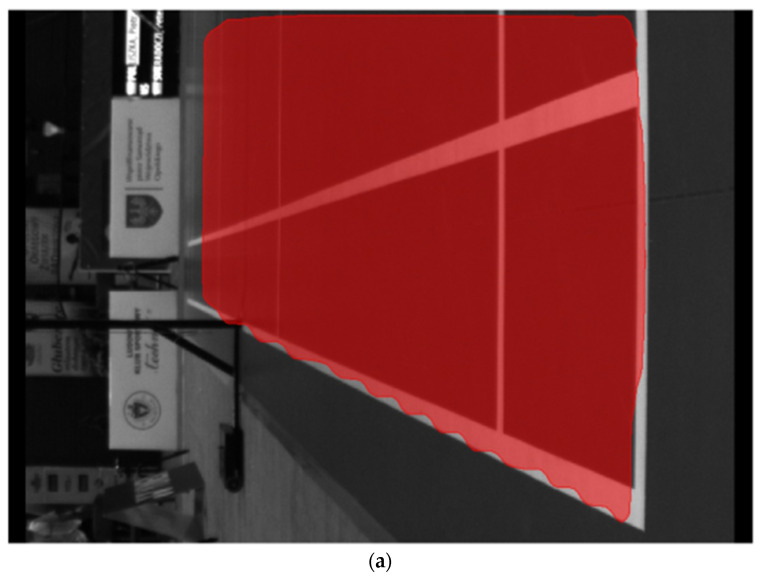

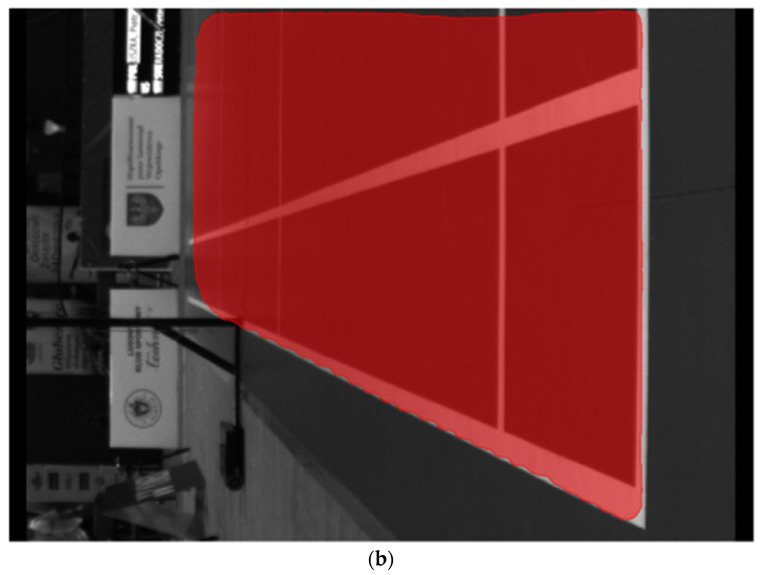
Masks of detected court: top, (**a**) original layers; bottom, (**b**) increased number of layers.

We twice increased the number of deconvolution layers in the mask detection subnetwork to achieve a higher resolution of the mask before rescaling it to the original image size. An example of generated masks with original (a) and increased (b) layers is shown in Figure 4 (notice the accuracy at the edges of the court). The model was trained partially on artificially generated images of the badminton courts. Original photos of the courts in our dataset were too similar, so they were filled with images generated from an artificial 3D court model. The accuracy of court detection is 97.7%.

### 2.9. Cameras Positions

Badminton regulations say that the distance from the court line to the closest object must be at least two meters clear space behind the baseline of the court (distance to any A-boards or similar advertising) and 1.5 m clear area from the sidelines of the court or between any two courts marked out side by side [29]. This rule is not always strictly followed. Often, organizers place courts closer to each other during the first days of a tournament. On the other hand, only one court is set up during the finals, and the distance to the advertising boards is much more considerable (cameras are placed behind them).

We mounted cameras below the ceiling and around the court in the first version of the system. In the current version, we use only cameras around the court.

The optimal camera positions for the best 3D tracking can be calculated by using the algorithm from Reference [30]. Unfortunately, the number of places where cameras can be installed in sports halls is so limited that using such algorithms is usually not applicable. Instead, we mounted at least four, and at most six, cameras to the hall’s ceiling. The four cameras must see the court from the four corners, and the additional two were usually placed on the court’s shorter sides.

The extrinsic parameters must be calculated for the cameras mounted above the court to track flying shuttlecocks in 3D precisely. There are many methods to achieve this. A technician can walk around the court with a board with markers. A single perfect sphere can replace a board [31]. A stiff, precisely measured stick with two perfect spheres can replace a single sphere for more accurate scaling. Instead of walking around the court, a technician can place flat markers on the court floor.

To simplify and accelerate the setup, we decided to use the whole court and intersections of the lines as markers. We base our method on ideas from Reference [25]. This calibration step is not needed for our current system version, as we now do not track shuttlecocks in 3D.

**Figure 5:**
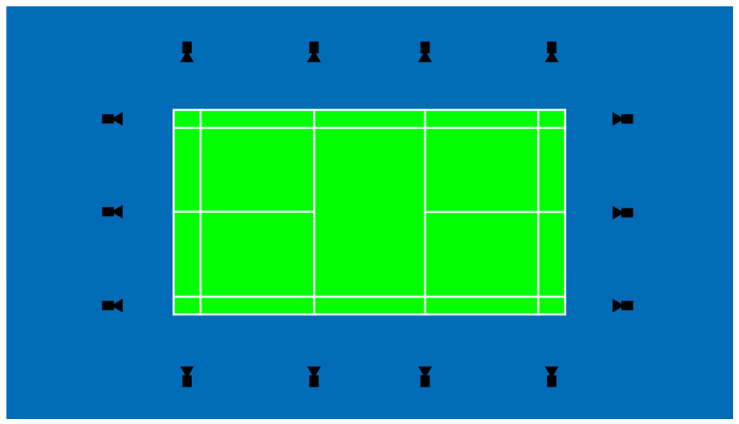
Cameras’ placement.

For the 14 cameras located around the court on tripods approximately 1 m above the ground, the placement is dictated by our tracking and ground touch detection algorithms (see Figure 5). Each line must be seen by a camera from each end to minimize occlusion.

**Figure 6:**
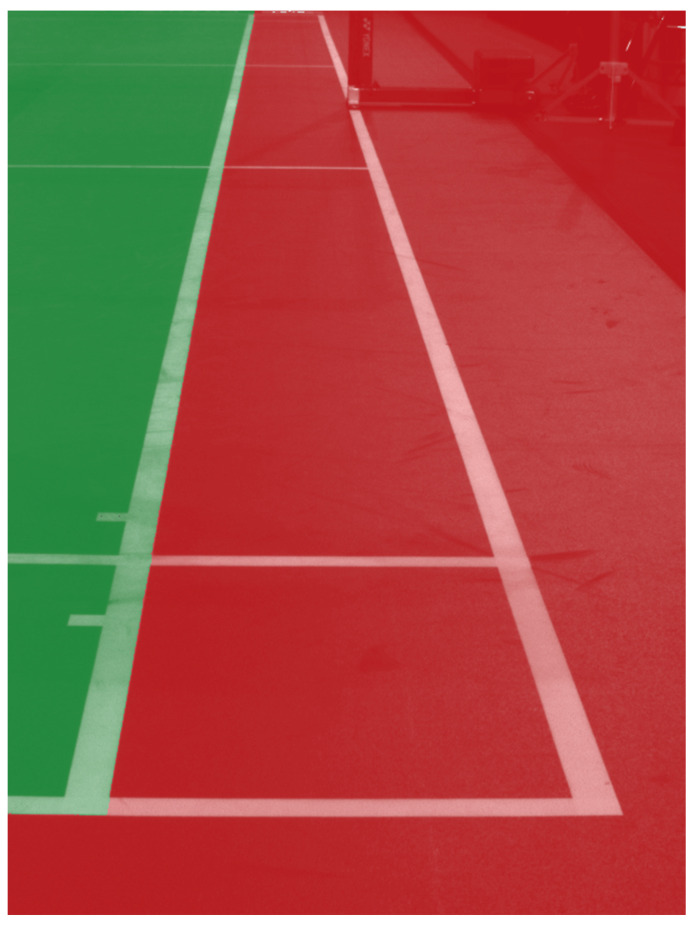
In and out area of a court. The green polygon represents the inside area, and the red is the outside (for the singles match).

Switching to 2D space allowed us to skip building a 3D model of a court and simplify camera calibration. We pass an image with only a sideline or back line of a court to the model and algorithm described in the previous section and divide the image into two polygons—outside and inside the court—as shown in Figure 6. The in/out decision is made by checking inside which polygon (red or green) the shuttlecock hit the ground.

### 2.10. Speed of Algorithms

High-speed cameras must be used to detect the exact moment of a ground hit. Our experiments show that 150–200 fps is the best compromise. That means there are only 5 or 6 ms for processing one frame image for the system to work in real time. Any sophisticated algorithms of image analysis that require a long computation time cannot be used. When we started working on our system, the fastest tracker based on the neural network was GOTURN [32]. We decided to develop a hybrid solution. It uses simple and fast detection and tracking methods and only sometimes uses a neural network on patches of single frames when the first method loses the tracked object.

### 2.11. Flickering Light

Algorithms based on differential frames are sensitive to flickering light. The newest and most modern sports halls are equipped with flicker-free lights, but most venues where national tournaments are played have old, flickering lights. We visited nine venues where badminton tournaments are organized, and only one had flicker-free lights installed.

Although many methods [33,34,35,36,37,38,39,40] and commercial software [41,42] remove flickering, they are not suitable for a fast video-stream processing. They are all designed for flicker removal’s effectiveness but not for execution speed. We concentrated on the speed with still reasonable and applicable results. We developed an adaptive pixel-wise method of generating masks that compensate for the flickering effect. Our method is 250–300 times faster than DeFlicker [41] and FlickerFree [42]. The method is described in detail in our paper [43]. We use the fact that our cameras do not move, as this allows us to calculate the similarity level of each pixel to the same pixel from the previous frame. If a pixel has changed because of a local movement in a scene, then the similarity level would be low. If similarity levels are higher than the threshold, then we interpret it as the flickering effect, which is reduced by our algorithm.

### 2.12. Shuttlecock Detection and Tracking

To be able to track any object, firstly, it must be found within the image. Many methods can be used for finding a specific object, from the most straightforward, such as color and shape segmentation, to methods utilizing neural networks. In Reference [44], the authors used the color segmentation method for detecting a tennis ball. In Reference [45], the authors, for each frame, generated a set of connected components by frame difference operation. All of these components are treated as ball candidates. Then the final decision is made by using a set of filters (object size, color, transparency). Xinguo Yu and Hon Wai Leong proposed in Reference [46] a two-phase trajectory-based algorithm that first generates a set of ball candidates for each frame and then uses them to compute the set of ball trajectories.

A similar approach, but instead using a shape-from-silhouette technique with multi-viewpoint images, was proposed by Shishido et al. [47].

In 2021, Zhiguang Cao et al. proposed two novel networks named M-YOLOv2 and YOLOBR based on Tiny YOLOv2 [48]. The authors modified the loss function to adaptively improve the detection speed for small objects, such as shuttlecocks and the architecture of Tiny YOLOv2, to retain more semantic information about small objects. Unfortunately, the neural network approach is slower than traditional methods. The method proposed works at most at 30 fps.

The use of a generic, universal approach for detecting shuttlecock is problematic because of the following reasons:

1. Depending on the camera’s view, badminton shuttlecock silhouette can be seen as a circle or a triangle, so finding it within the image is more complex than finding a ball.
2. The size of the shuttlecock may significantly change when it approaches the camera.
3. A shuttlecock moves at rapidly varying speeds, reaching very high values at times. The algorithm must work well with fast (400 km/h) and slow (24 km/h) shuttlecocks.
4. The white color of the shuttlecock can be very similar to the scene (player socks, white court lines, and letters on advertisement boards).
5. The shuttlecock often moves in complex contexts: moving players, fast rackets, and changing backgrounds.
6. Because of the cone-like shape and center of gravity located next to the cork (not in the center), it is difficult to predict the trajectory following the physics laws described by simple formulas, especially after the hit, when the shuttlecock turns over and has an unstable trajectory.

In order to avoid as many problems as possible, in our current system version, we located cameras next to the court (behind advertisement boards), facing the lines. We do not use cameras over the court. This approach has one limit: we do not record the whole trajectory, but it is sufficient for in/out decisions.

By narrowing the field of view of the camera to the area of the field line, we limit the visibility of other moving objects (players, for example); thus, the shuttlecock is very often the only moving object. That also simplifies the problem with unstable trajectory: it is stable next to the ground. Moreover, the speed of a shuttlecock is almost constant.

We utilize the feature that a shuttlecock is a fast-moving object over a usually still background. Differential images generated from consecutive frames allow us to distinguish moving objects from static backgrounds.

**Figure 7:**
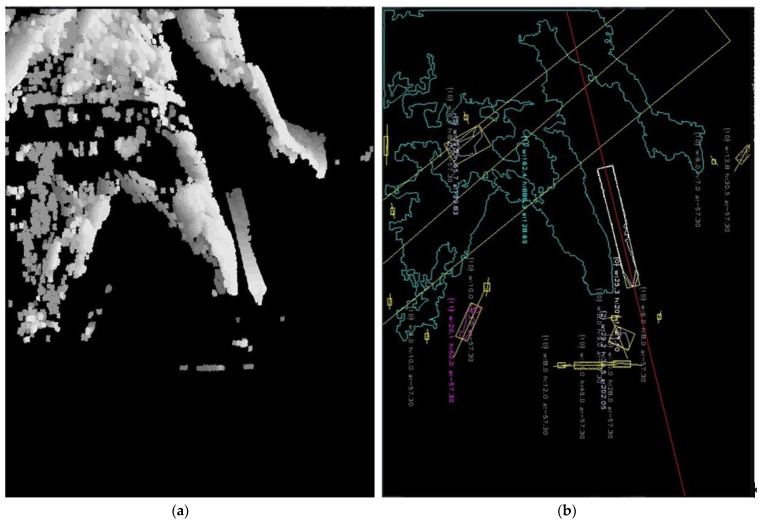
Accumulative differential frame (**a**) (on the **left**). Blobs with marked moving direction by lines (**b**) (on the **right**). The white blob is the one that was marked as a shuttlecock. The color of a blob represents the filter that filtered out the shuttlecock candidate.

On the other hand, when the shuttlecock hits the ground, it changes direction and may even stop moving for a moment and vanish from the differential image. Our solution generates an accumulative differential frame from 7 consecutive differential images. This allows us to keep sight of the shuttlecock when it hits the ground. The intensity of pixels in the accumulative frame corresponds to the order of images used to generate it. Pixels from later differential images obtain lower values than pixels from earlier images (from light to a dark shade of gray color). This way, we get additional information about the direction of the movement. Figure 7 shows an accumulative differential frame (a) and the direction of moving blobs marked by lines (b). The blob with the white outline in Figure 7b is the one that was recognized as a shuttlecock. Other blobs in this figure were filtered out. The color of a blob represents the filter that filtered out the shuttlecock candidate.

Similar to Reference [45], our solution generates a set of shuttlecock candidates (blobs) and, using a set of filters, selects the one most likely to be a shuttlecock. The filters are as follows:

1. Remove big blobs (players)—marked with aqua color on Figure 7b.
2. Remove small blobs (noise)—gray color.
3. Remove blobs whose shape is not pencil-like—magenta color.
4. Remove blobs that move too slow or too fast—red color.
5. Remove blobs that are moving in the wrong direction (pink color). In our scenario, we are interested in tracking the shuttlecock when it moves down to the ground.

Shape segmentation makes it easy to distinguish a shuttlecock from another moving object. The whole process takes less than 2 ms on an Intel Core i7 machine.

**Figure 8:**
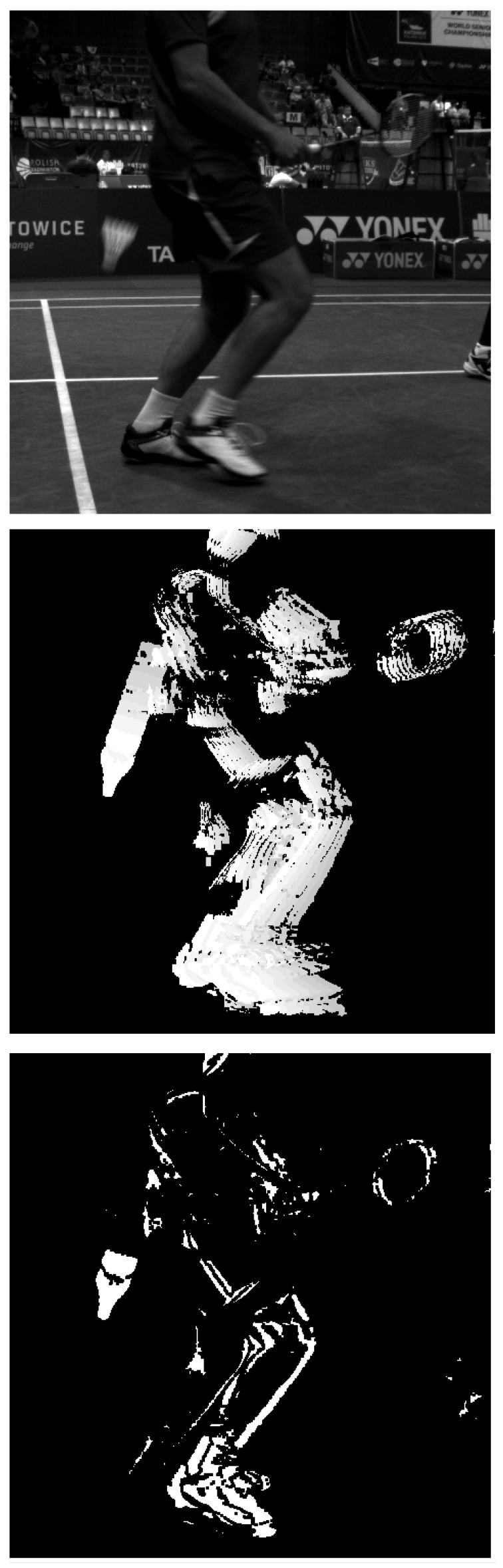
Shuttlecock moving along a player: top = original image, middle = accumulated diff, and bottom = differential image.

**Figure 9:**
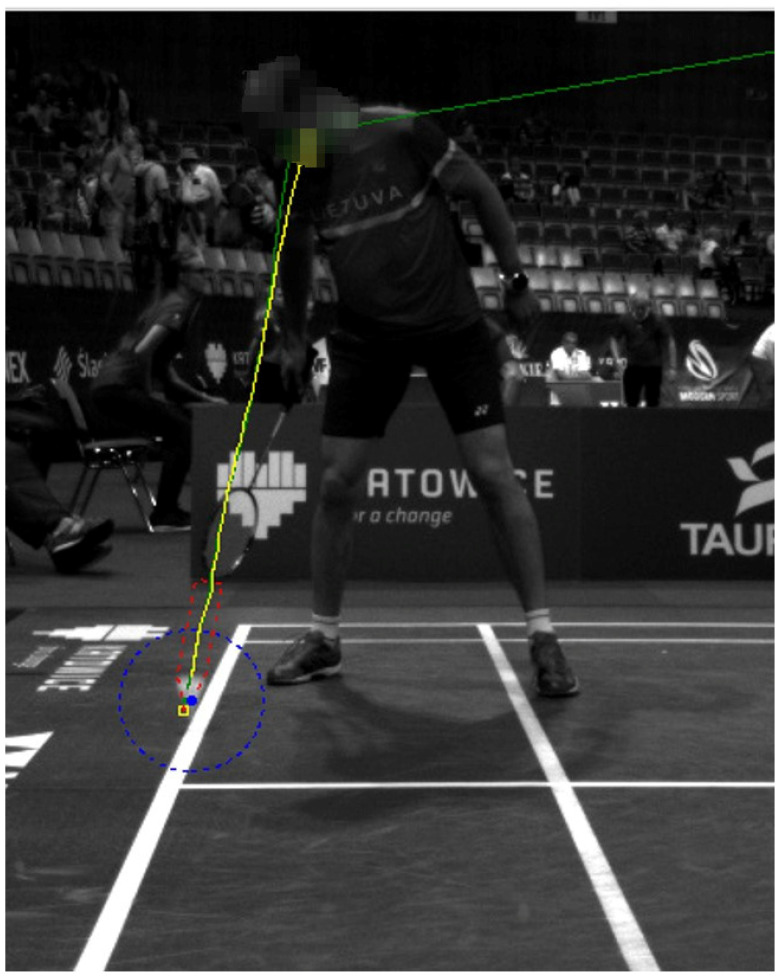
Predicted position of a shuttlecock by using Kalman filter (green color); ground-truth position marked by the operator (yellow color).

There are situations when, behind a shuttlecock, a player is moving. The blob of a player is usually much bigger than the blob of a shuttlecock, and both blobs overlap. The segmentation algorithm may mistakenly treat the shuttlecock as a fragment of a player. Figure 8 shows an example of this problem. To solve it, we follow the algorithm:

1. With the use of a Kalman filter, predict where the shuttle should be, based on the trajectory from the previous frames (Figure 9).
2. Generate a patch (104 × 104 px) from the original frame with the center predicted by a Kalman filter.
3. Pass a generated patch to a neural network detector as an input.

We perform this additional detection only if the first part of the detector fails.

**Figure 10:**
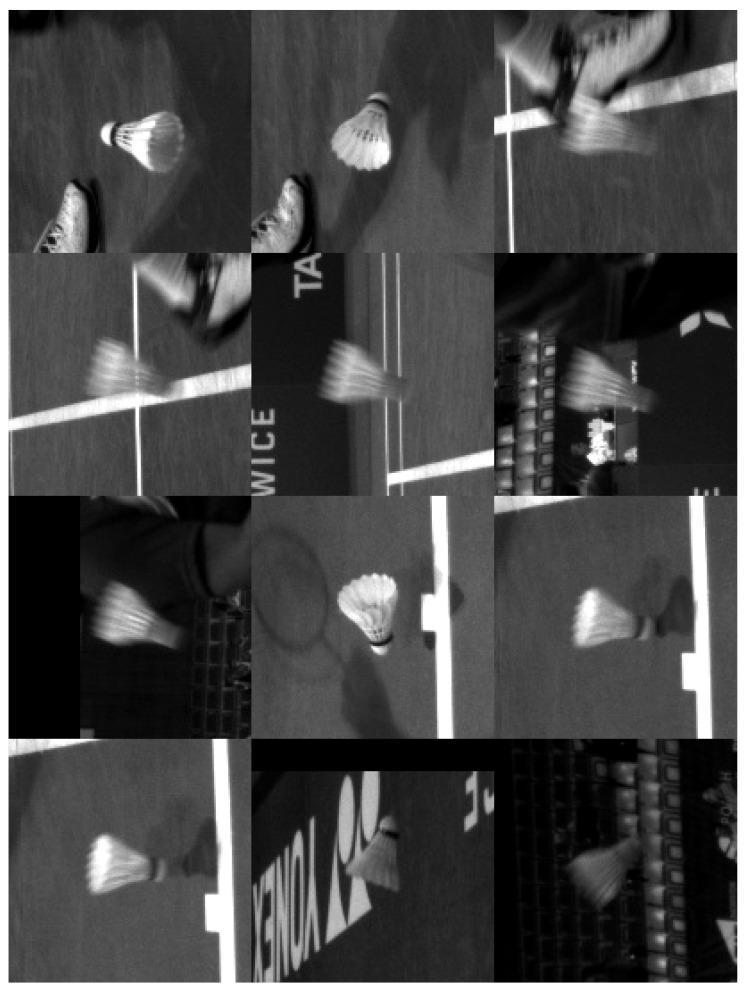
Example of images passed to the neural network.

**Figure 11:**
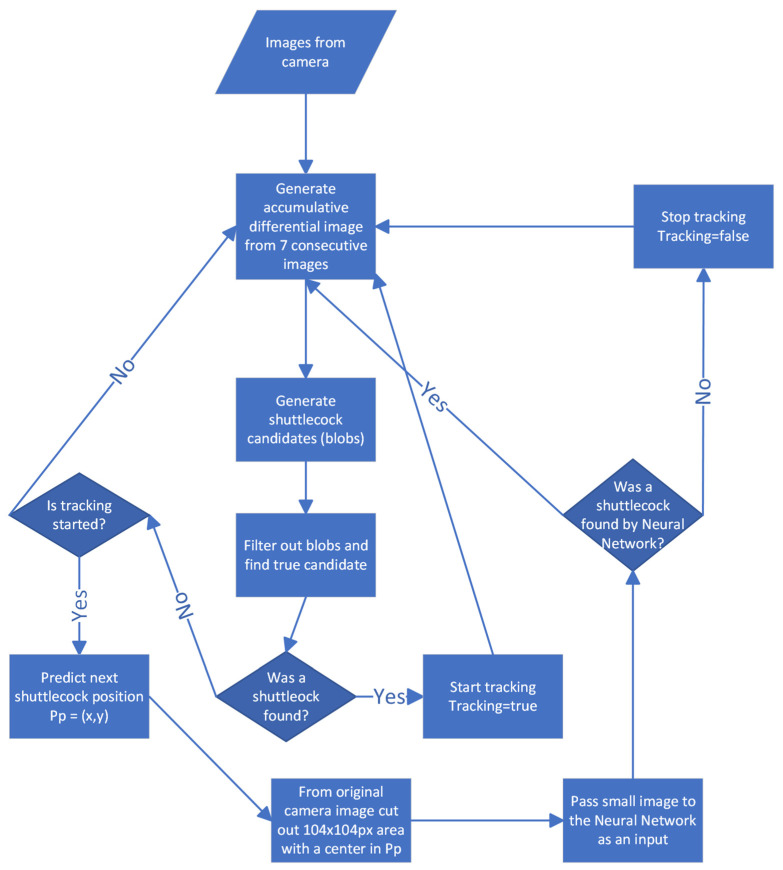
Algorithm structure.

Our neural network is a modified Tiny Yolo3 [49] trained with a set of images collected during the tournaments. The original Tiny Yolo3 expects 3-channel photos, and we changed it to use only 1-channel ones. Figure 10 presents example patches passed to the neural network. With the CPU: 9th Gen. Intel^®^ Core™ i7-processor and GPU: NVIDIA^®^ GTX 1080, the detection of a shuttlecock with a neural network takes 12 ms and causes a delay, which is, however, acceptable. When the first method finds the shuttlecock again, it eliminates the delay caused by the neural network, and the entire algorithm works in almost-real time. The neural network with a single class (shuttlecock) achieves a mean average precision (mAP@0.50) of 94%, and for a threshold 0.25 precision, it achieves 0.96, a recall of 0.77, and an F1-score of 0.86. Figure 11 shows the algorithm’s structure. Two videos with the result of our algorithm are available at https://drive.google.com/file/d/1rsvcY-5Q25iN5Hmcv4nW_DXIWq9ZAR7J/view?usp=sharing, https://drive.google.com/file/d/1-Btu3YHeyYYm29MncjXMvjHcUePi2EI_/view?usp=sharing (accessed on 16 October 2022).

### 2.13. Tracker Performance

We collected data for the tests during the tournaments. Each time the player disagreed with the line judge’s decision and asked for verification, our system saved 30 s of camera footage at 150 fps. From recorded movies, we selected 52 sets of frames for annotation. Sets of images and the file with annotated shuttlecock positions are available to download from the location provided in the Supplementary Materials Section. Each set consists of several consecutive frames before the shuttlecock hits the floor. We annotated a total of 20,788 frames. If the shuttlecock was visible, the operator marked its center position. More precisely, the operator marked positions of only a few frames where the shuttlecock was visible, and we interpolated the rest of the positions between those marked.

Our tracker detects if the shuttlecock is visible with an accuracy of 94%. We prefer false detections to missed, undetected shuttlecocks. These can be socks, shoes, and other white parts of clothing.

For the frames where the shuttlecock was visible, we measured if the detected position of a shuttlecock was correct. Due to the irregular shape of the shuttlecock, inaccuracies in interpolation, and blurriness, the shuttlecock’s annotated positions are not always accurate. The detection of a shuttlecock position was marked successful if the difference between annotated position and the position saved by the detector was less than 12 pixels. In all, 81% of the visible shuttlecocks were correctly detected and tracked.

### 2.14. Object Segmentation

While the shuttlecock moves at a high speed, it is blurred within the image. The motion blur effect causes segmentation difficulties due to ambiguous pixels between regions of the object and the background. Imprecise shuttlecock contour detection reduces the accuracy of localizing the cork when it comes into contact with the ground impairing the overall system’s accuracy.

**Figure 12:**
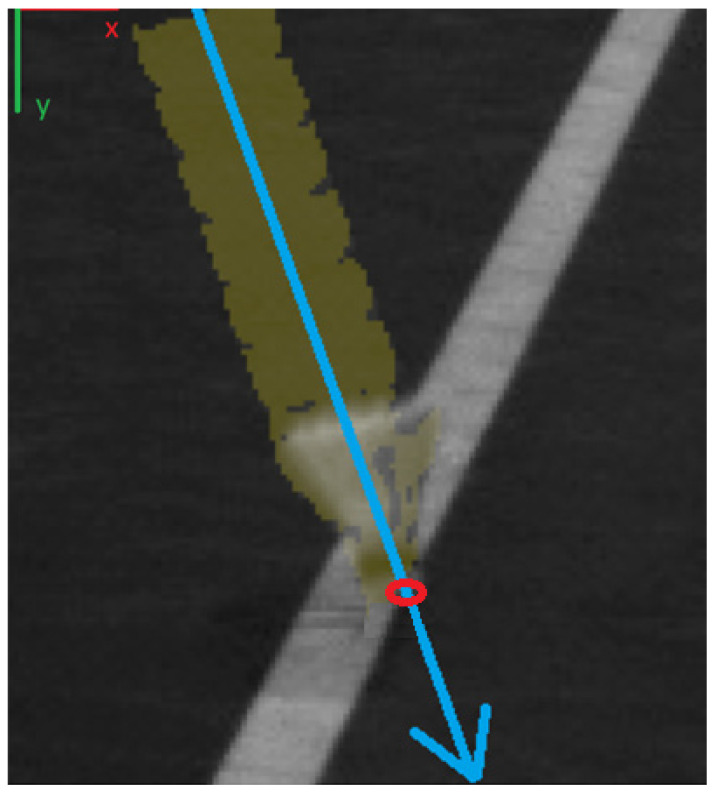
Accumulated diff (yellow) together with an original image. Red circle = ground touch point. Blue arrow = movement direction.

Although there are many methods to remove or decrease blur (see, for example References [50,51,52]), we do not use them. Instead, we take advantage of the symmetry of the moving shuttlecock. Just before it hits the ground, its trajectory is an almost perfectly straight line. The symmetry line of the shuttlecock in the accumulative differential frame indicates the cork before and just when the shuttle hits the ground (Figure 12).

**Figure 13:**
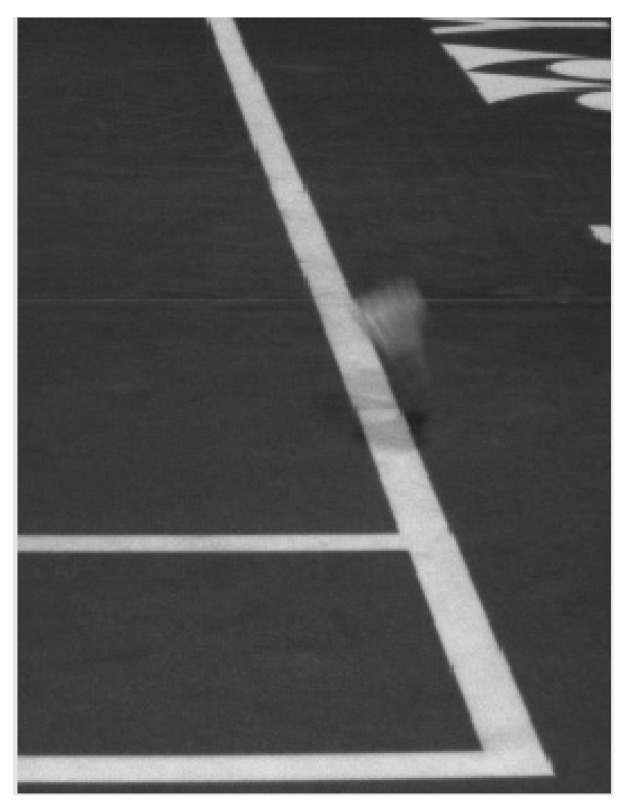
Before the ground hit.

**Figure 14:**
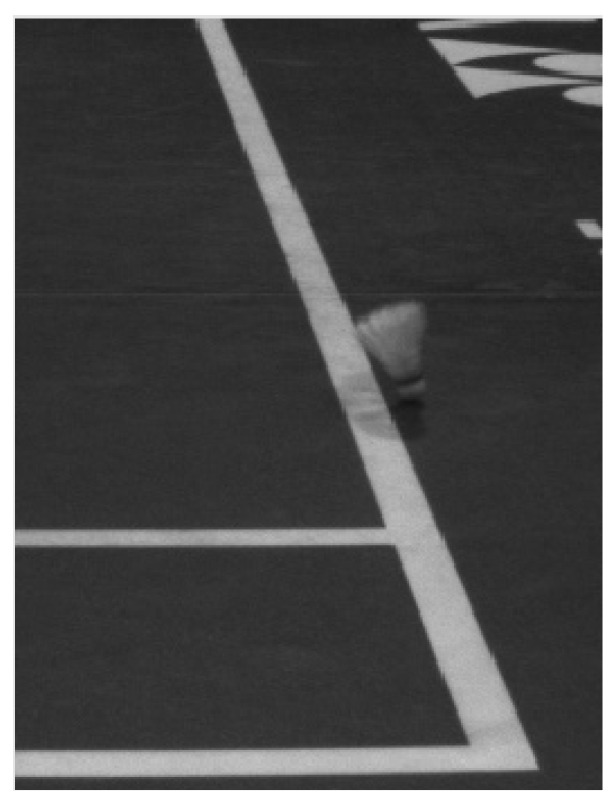
Ground hit.

**Figure 15:**
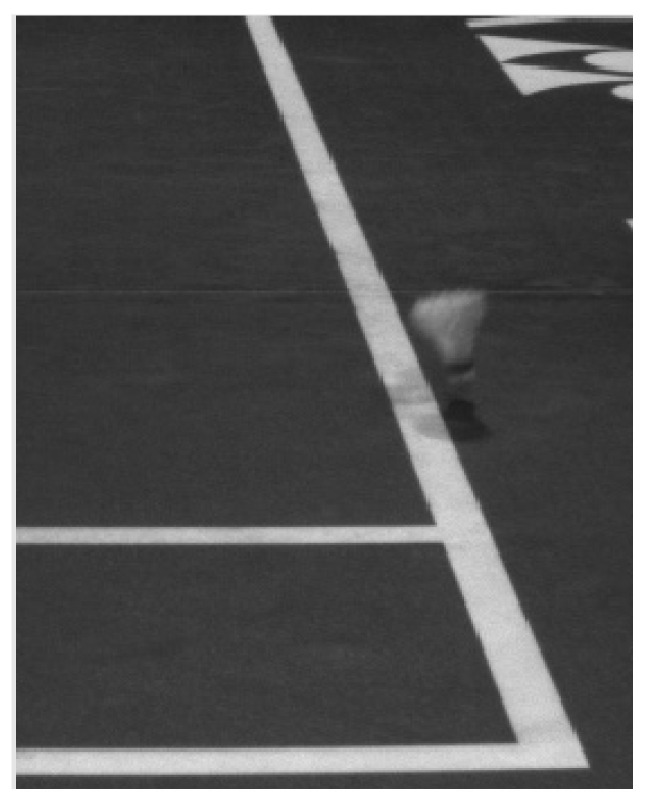
After the ground hit.

When the shuttlecock hits the ground, it usually slows down. The exception is when the shuttle flies almost horizontally and slides on the ground. The sudden change in speed is also evident in the change in the degree of blurring (see Figure 13, Figure 14 and Figure 15).

### 2.15. Finding the Frame When the Shuttlecock Hits the Ground

**Figure 16:**
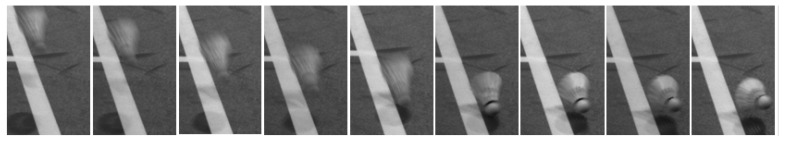
Example shuttlecock trajectory before and after ground hit.

The standard approach for finding a ground hit is a trajectory change. It works well for tennis, where the ball is resilient, and the bounce is high. Shuttlecock does not bounce very high, and sometimes it may even slide, so the trajectory does not change significantly. Figure 16 shows a set of images of a shuttlecock before and after the ground touch. As can be seen, the speed of a shuttlecock changes more significantly than the trajectory. That is why, except for using trajectory-change information, we also utilize the shuttlecock blurriness measure calculated as the variance of the Laplacian. That value rapidly changes when the shuttlecock hits the ground.

### 2.16. Tracked Object Occlusions

A player may occlude the shuttlecock when it approaches the ground. We position cameras on both sides of each court line to solve this problem. Then, in most cases, at least one camera sees the shuttlecock when it hits the ground. Moreover, from data collected during the competitions, we learned that, usually, there is at least one more camera that sees the ground touch from a different angle (the camera that observes the nearest perpendicular line). All cameras are synchronized and record frames in identical timestamps. When possible, we use frame sets from all cameras that see the shuttlecock to determine the frame in which it hit the ground. If the results differ, we use simple voting and warn the operator, who may quickly verify and change the automatically selected frame.

After determining the ground touch moment, only frames from cameras located at the ends of the line can be used to decide on which side of this line the shuttle hit the ground. Usually, only one camera is used, i.e., the one closer to the shuttlecock. In the case of occlusion, the other farther camera is used. There are two worst scenarios, both for the sidelines. The first is when two players on opposite court sides occlude the view of cameras on both ends of the line. The second is when only one player occludes the view of the closer camera, but the shuttlecock is at the end of the line. The other camera is far from the shuttlecock and has a lens set to observe closer objects. The combined court length and the camera’s distance to the court may be 20 m. The moving shuttlecock recorded from this distance may be too small to decide where it landed.

For other lines, the first problem may happen for doubles, but the second one does not exist because all the other lines are much shorter than the sidelines.

If the system cannot verify the ground touch location, it gives up, and the judge’s first decision remains upheld. The players are informed that verifying the linesman’s decision is impossible.

## 3. Results

The system presents the results in the form of an artificial animation. Such a solution is attractive to the audience and prevents discussions and doubts when the original camera image is blurred or the shuttlecock is partially obscured. The examples of our challenge animations from tournaments can be seen in Reference [53].

We provided judges with a series of images based on which the system has made a decision. We do it both during the challenge call (we show images on the umpire’s tablet) and after the match (we provide the recordings in a report).

Our system delivers a correct decision in 62% of cases. Even a tiny error in one of the steps of the processing pipeline, i.e., shuttlecock detection, shuttlecock tracking, ground hit frame detection, line detection, cork segmentation, and in–out decision, may lead to the wrong system’s answer. That is why, in our system, the human operator confirms the final in/out decision to avoid such situations as during Wimbledon 2022 [54] or DAIHATSU Indonesia Masters 2021 [55] when the players strongly disagreed with the Hawk-Eye decision. It is hard to compare our results with other systems because other companies do not share their data. Our data are available to download from the location provided in the Supplementary Materials Section.

When the player disagrees with the linesman’s decision, he asks the umpire for verification by a system. If the umpire agrees to a challenge, he raises a hand and calls a challenge. The operator seeing the raised umpire’s hand starts the automatic procedure in our system. Almost immediately, the system presents the decision and additional data: a series of images with added marks and visual hints indicating where the system found the shuttle, the cork, and which frame (or pair of frames) was selected as the moment of ground touch. The operator verifies the data and the result. He may fix the results of every stage of the system’s pipeline. For example, he can decide that the ground touch happened two frames earlier than indicated by the system, or he can adjust the location of the detected cork. After each manual fix, the following part of the pipeline is recalculated. Finally, the operator accepts the decision. The animation is generated, shown to the audience, and sent to the TV broadcast.

We put much effort into making this procedure as easy and fast as possible. Now it usually takes from 7 to 25 s.

Table 4 presents all the system’s decisions from one court during the World Senior Championships played in Katowice. Most of the automated decisions did not need to be modified by the operator.

Players and spectators wait for the decision for 18 s, on average. This time is not only acceptable but desirable. A shorter waiting time would reduce audience emotions, and a longer would make spectators impatient or even bored.

Our data clearly show that linesmen in 51 out of 211 (24.2%) cases make wrong calls, and a system like ours is needed to make the game fair.

Compared to the data collected by Primo L. et al. [56] during the Olympic Games (Rio de Janeiro, 2016) and the Word Badminton Championship (Glasgow, 2017), where judges were wrong in 11 out of 56 calls (19.6%), judges in Katowice made more wrong decisions than during the elite tournaments. In our opinion, this is because judges selected for the top elite tournaments were much more experienced than judges who worked in Katowice.

## 4. Discussion

Our system was approved for the tournament of the Senior World Championships in Katowice, organized by the Badminton World Federation (BWF). It is certified by the Polish Badminton Association (PZBad) and was also verified by Badminton Europe (BE).

The BWF verification procedure was based on a system test run in the “blind” mode: players could not use it. However, the system checked all questionable decisions of line judges, and a dedicated judge randomly verified the results of the system. After one day of such a procedure, the system was released for use by players during the tournament. PZBad issued us a certificate based on the opinions of judges who used the system during several tournaments in which our system was tested. BE sent a representative to one of the tournaments, where we tested the system. The BE representative observed the system in action during the tournament, interviewed the judges after the tournament, and analyzed the possibilities and how the system worked during the technical workshop we organized for him. Neither of these procedures took into account the objectively measured accuracy of the system.

We did not conduct any official error-measurement procedures because the Badminton World Federation does not have official evaluation procedures yet. International Tennis Federation has one [57], but the procedure does not say how to measure the system’s accuracy precisely. The ITF procedure says that “high-speed video cameras are used as the sole and definitive method by which accuracy is established. The “mark” left on the court surface following impact is used only to aid in the selection of impacts to analyze”. We could record the shuttlecock hitting the ground with faster cameras with higher resolutions than ours and compare system decisions with the recordings. Unfortunately, for the faster cameras, we would need much more light and some changes to our algorithms, which are calibrated to different lighting conditions.

Instead of faster cameras, we could experiment with powder on the floor, but we did not, and we performed an accuracy test by using footage from the cameras we are using in our system. The human operator, analyzing images, decided if the shuttlecock was in or out. This decision was compared with the response from the system.

Our system is not perfect. Sometimes, significantly when the shuttlecock interferes with the racket or the player occludes the shuttle, the system fails to detect the moment of the ground touch accurately. Fortunately, it is designed so that the operator can rapidly fix such minor errors.

When the Hawk-Eye system passed ITF testing measures in 2007, the declared mean error was 3.6 mm [58]. We did not find any information about the accuracy of the Hawk-Eye system for badminton. Most likely, it will be different because the shuttle is an object that is more difficult to track than a tennis ball. Moreover, we do not know how exactly the Hawk-Eye system was tested. Did the test procedure include interfering players and rackets?

We compared the opinions of the judges that used both systems, response times, and percentages of successful challenge calls. They all are similar. For example, in the video available on YouTube from the fifth day of the World Seniors Championships 2019, there were 16 challenges verified by our system. The first two are long (58 and 35 s), but the rest of the times are very similar to the 25 times of the Hawk-Eye challenges during 16 matches of the World Championships 2021, also available on YouTube. The averages are 18.43 s (ours) and 18.00 s (Hawk-Eye). Surprisingly, both systems showed that the linesmen were wrong in the same percentage of the cases (21–24%). We believe that both systems have similar accuracy and average time to show the decision. Our contribution is a novel architecture without cameras above the court and algorithms that can detect the place of the ground touch only from 2D images. In our opinion, this new architecture has important practical implications compared to the Hawk-Eye: less hardware (cameras and cables), cheaper transport, and faster and easier installation. More accessible cameras (no cameras at the ceiling) make the system less prone to errors. This setup is also more adaptable, elastic, and universal in different sports halls.

We highly recommend an analysis of the public understanding of Hawk-Eye and similar technology in cricket and tennis by Harry Collins and Robert Evans [59]. We agree with the authors about all the doubts about accuracy, errors, and approval procedures. We disagree with some of their conclusions and proposals. First, unlike Collins and Evans, we believe that the system should preset the decision without any information about certainty or probability of mistake. The purpose of artificial animation is the same: show that there is no doubt and no space for discussion or interpretation. The challenge system displays the final, indisputable decision which competitors and judges must follow. Second, the authors suggest: “[…] technological decision aids should be adjusted to make the same systematic errors as are made by existing human judges so the game changes as little as possible”. In badminton, there are at least two aspects where it could be applied: the size and shape of the area touched by the shuttlecock and the belief that the shuttlecock always touches the ground with the cork first.

The diameter of the cork is 26–28 mm. It is hard and is unlikely to deform. We assumed that the contact surface of the cork with the ground is a perfect circle with a diameter equal to 13 mm. The Hawk-Eye system works the same. In reality, the air temperature, the flexibility of the mat, the speed, and the flight direction of the shuttlecock may influence this area. Moreover, players and linesmen see the shuttlecock not from the ground level but from above and tend to treat the whole cork’s diameter as the diameter of the touch area.

The shuttlecock hit hard just above the net in the direction of the court’s end line will touch the ground with the feather first. All of the judges we spoke to were surprised by this fact. Linesmen make decisions based on the position of the cork and not the feathers making big mistakes. Fortunately, this is a rare situation. We believe that our system should not copy these errors.

## 5. Further Work

The main challenge is to improve the accuracy of the system. Accuracy can be increased by using higher-resolution cameras or increasing their number.

The capabilities of hardware available on the market change over time: The newest cameras have the same speed but much higher resolution than the ones we bought a few years ago. They are also much more expensive.

Buying better cameras is not enough, because higher resolution means that our system must process much more data at the same constrained time. Increasing the computing power of computers must be combined with changes in algorithms to adapt to much larger data streams.

It should be noted that the camera’s high resolution is only needed when the shuttlecock hits the ground. A higher acquisition speed is needed to determine the moment at which the shuttlecock rebounds against the ground. A higher resolution can also be obtained by using super-resolution algorithms, which may be an element of further investigation.

Since the automatic decision whether the shuttlecock falls in the field of play or outside of it must be presented within 10–25 s, the equipment and algorithms should be wisely selected.

We have tools that monitor connectivity with cameras, but this is not the only risk that needs to be monitored. During the tournaments, on several occasions, the camera was touched by persons who were walking around the outside court area (especially the TV crew). Now system operators control such situations manually. Automatic camera-displacement detection is not a complicated problem, and it would ease system operators’ work.

In the future, we plan to improve our algorithms to achieve better accuracy in detecting the moment of contact with the ground. It should shorten the average time to present the decision from the current 18 s to 12 s (our goal).

## 6. Conclusions

Our four-year work has resulted in developing an application that supports line judges during badminton tournaments and checks whether the shuttlecock has landed on or off the court. We had to deal with many problems, even the ones we did not expect to happen (flashing lights and no room to place cameras at the proper distance from the line).

Our system was used during several badminton tournaments, including the world senior championship.

In this study, we presented how a seemingly uncomplicated thing—an automatic assessment of whether the shuttlecock has fallen inside or outside the court—requires solving several problems that are hard to predict in the laboratory but occur under real conditions and significantly impact the usefulness of the developed solution.

To measure the system’s accuracy, we used the data from real tournaments only when a player disagreed with the linesman’s decision. The system’s overall efficiency is not high enough to dispense with human verification of the system’s response by analyzing slow-motion video. However, this was not our goal. The proposed system only serves the role of supporting the essential function of a judge by analyzing and explaining the observed circumstances in disputes.

Thanks to the developed software, even if a human makes the final decision, it is consistent with reality and makes the game result fair.

Since we play badminton ourselves and know many judges and players, we had the opportunity to test our ideas and algorithms in real conditions at a very early stage of work. Thanks to this, we knew whether we were going in the right direction. Our goal was not only to conduct scientific research but to build a working application.

Because the computer equipment available on the market has higher efficiency and the cameras have better quality each year, improving the developed system may be possible. The use of neural network models is promising, but we wait for the day when they will operate at least 200 fps. A more stable, better quality time-resolution image will undoubtedly allow for greater precision tracking of the shuttlecock. However, a qualitative improvement in the performance of the detection system will be possible only when the conditions under which the competition is conducted (lighting, air-conditioning, changes in temperature and shuttlecock parameters, court quality, etc.) are normalized and stabilized.

## Figures and Tables

**Table 1:** Primary camera interfaces.

	Distance (m)	Bandwidth (Gbps)
CoaXPress 2.0	100	12
CameraLink	5 (10 in theory)	2 (4 with 2 cables)
USB 3.0	3	5
GigE	100	10

**Table 2:** Tested sensors.

Sensor	Sony IMX531	Sony IMX255
Sensor Format	1.1″	1″
Sensor Type	CMOS	CMOS
Resolution (H × V)	4512 × 4512 px	4096 × 2160 px
Resolution	20 MP	8.9 MP
Pixel Size (H × V)	2.74 µm × 2.74 µm	3.45 μm × 3.45 μm

**Table 3:** How increasing gain affects camera noise.

Sensor	Gain (dB)	SNR (dB)	PSNR (dB)
Sony IMX255	0	30.92	42.24
	+6	29.91	37.91
	+12	26.32	35.57
Sony IMX531	0	32.75	36.22
	+6	30.18	32.08
	+12	26.71	30.52

**Table 4:** Distribution of successful and unsuccessful challenges.

Scenario	Number of Challenges
Challenges unsuccessful	160
Challenges successful	51
TOTAL	211
Which line was verified	
Side lines	109
Base lines, long service lines	75
Short service lines	20
Center lines	7

## Data Availability

The data presented in this study are available online at https://drive.google.com/drive/folders/1cacjO6hqkgT8-o-1BfPn-__UgiCaOLyF?usp=sharing (accessed on 16 October 2022).

## Supplementary Materials

The following supporting information can be downloaded from the following links: https://drive.google.com/file/d/111KPyoUqMMw50ElyeIx0_77lt_WSw2qW/view?usp=sharing (Data annotation file); https://drive.google.com/file/d/1iEQ7dC9g1pWLXzF7PnToB1V8FIzVuzDS/view?usp=sharing (Sets of images); https://drive.google.com/file/d/1rsvcY-5Q25iN5Hmcv4nW_DXIWq9ZAR7J/view?usp=sharing and https://drive.google.com/file/d/1-Btu3YHeyYYm29MncjXMvjHcUePi2EI_/view?usp=sharing (video presenting the result of tracking and segmentation algorithm).
